# Cataracte sénile avec capsule antérieure crétacé

**DOI:** 10.11604/pamj.2015.20.85.4505

**Published:** 2015-01-29

**Authors:** Jaja Zineb, Lazrek Omar

**Affiliations:** 1 Service d'Ophtalmologie A, Hôpital des Spécialités, CHU Rabat, Rabat, Maroc

**Keywords:** Cataracte, sclérose du nucléus, opacification, cataract, sclerosis of the nucleus, opacification

## Image en medicine

La cataracte est l'opacification partielle ou totale du cristallin. Le type principal en relation avec l’âge est une sclérose du nucléus, corticale, et postérieure sous-capsulaire. Les opacités da la capsule donnent un aspect offrent souvent une surface lisse et polie quelque fois au lieu de cet aspect elles presentent un aspect crétacé (couleur de craie et aspect dépolie).

**Figure 1:**
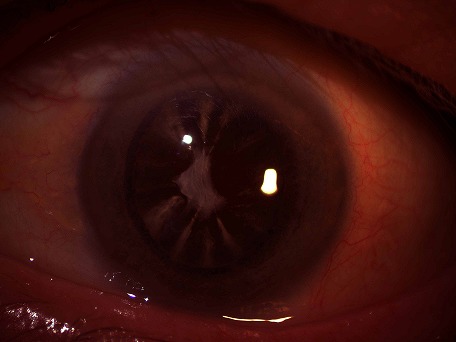
Aspect de capsule antérieure crétacé

